# Supplementary material for: Trojan Horse nanotheranostics with dual transformability and multifunctionality for highly effective cancer treatment
Source: Nat Commun. 2018 Sep 7;9:3653. doi: 10.1038/s41467-018-06093-5 (PMC6128896; doi:10.1038/s41467-018-06093-5)
Supplement: Supplementary file 1 — Supplementary Information [file 41467_2018_6093_MOESM1_ESM.pdf]

Supplementary Information

Trojan Horse Nanotheranostics with Dual Transformability and Multifunctionality for Highly Effective Cancer Treatment

Xue et. al.

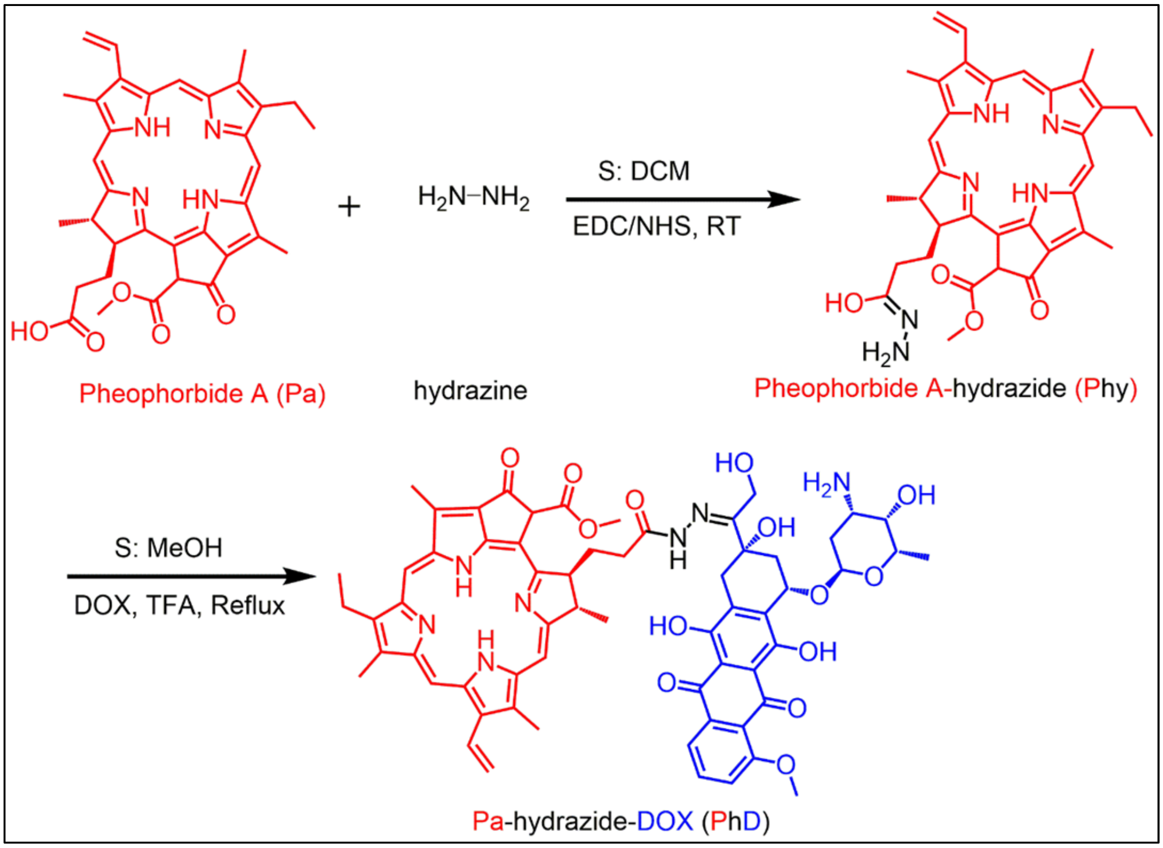

Supplementary Figure 1. Synthetic route of PhD monomer. Pheophorbide a (Pa) firstly reacted with hydrazine to form pheophorbide a-hydrazide (Phy). The Phy then conjugated with doxorubicin (DOX) through hydrazone bond to form Pa-hydrazide-DOX monomer (PhD).

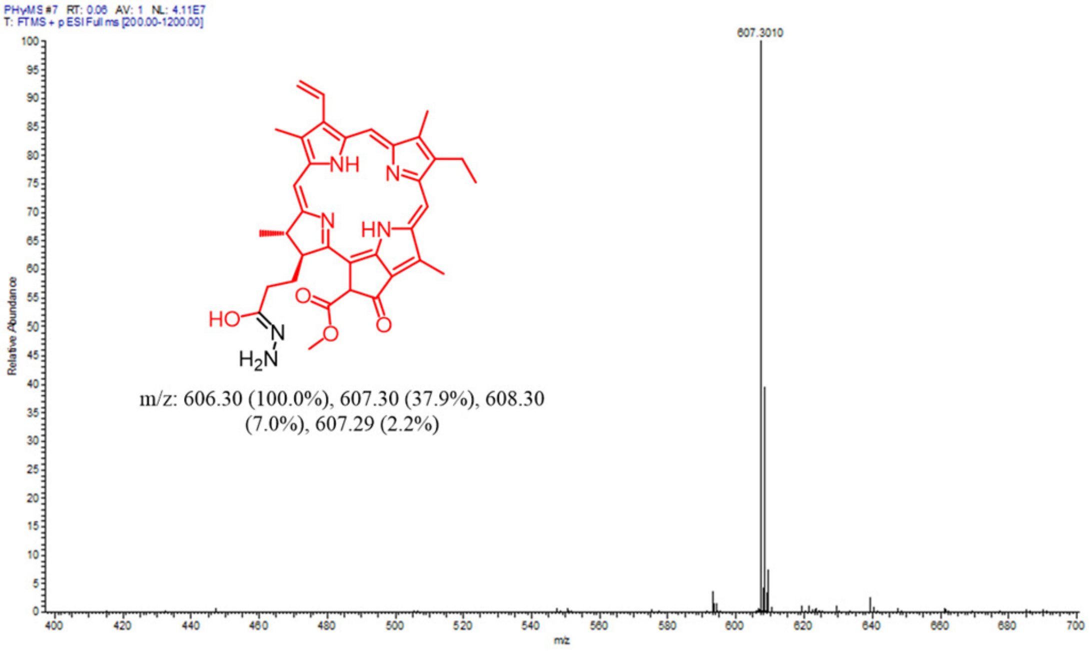

Supplementary Figure 2. High-resolution mass spectrometry (HRMS) analysis of molecular weight of Phy. *m/z* 607.3010 is the M+$[H]^+$ peak.

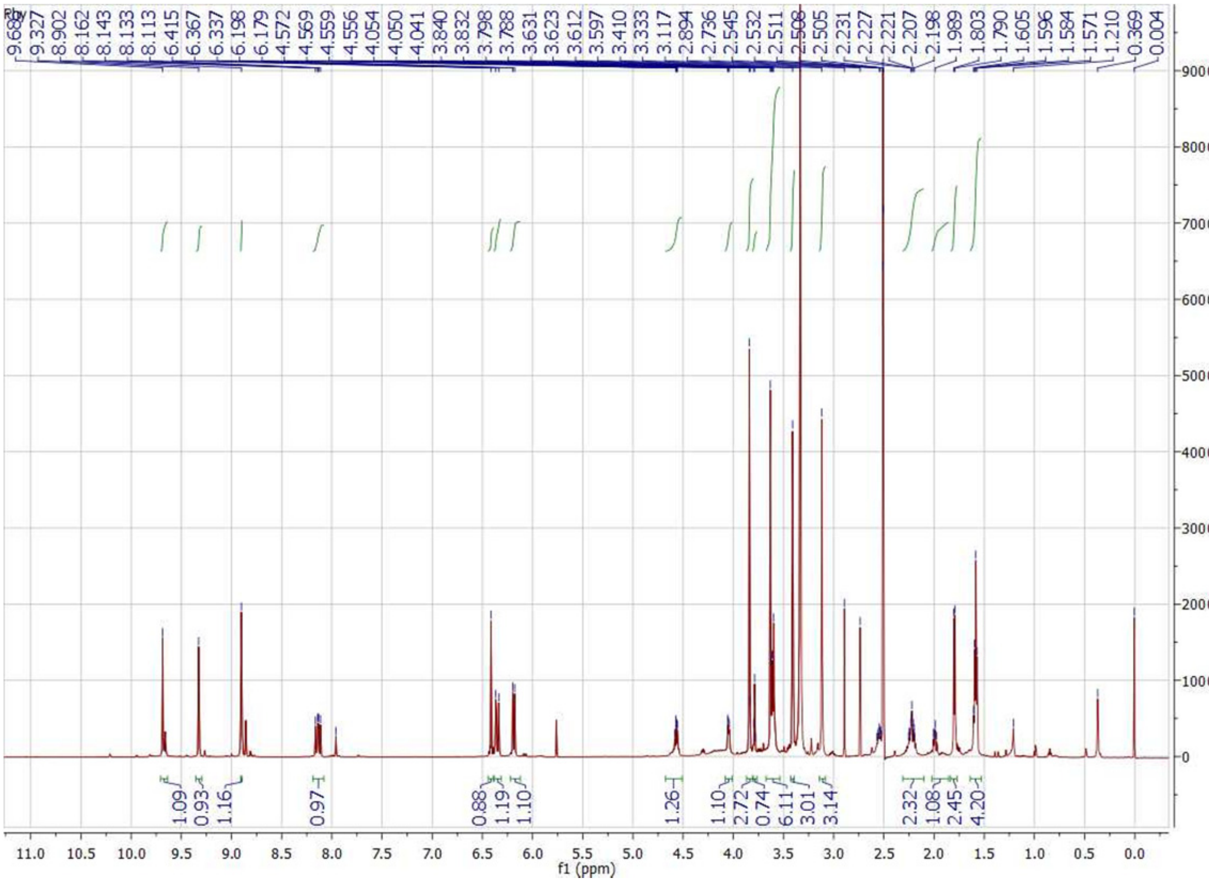

Supplementary Figure 3. ^{1}H-NMR (nuclear magnetic resonance) spectrum of Phy monomer.

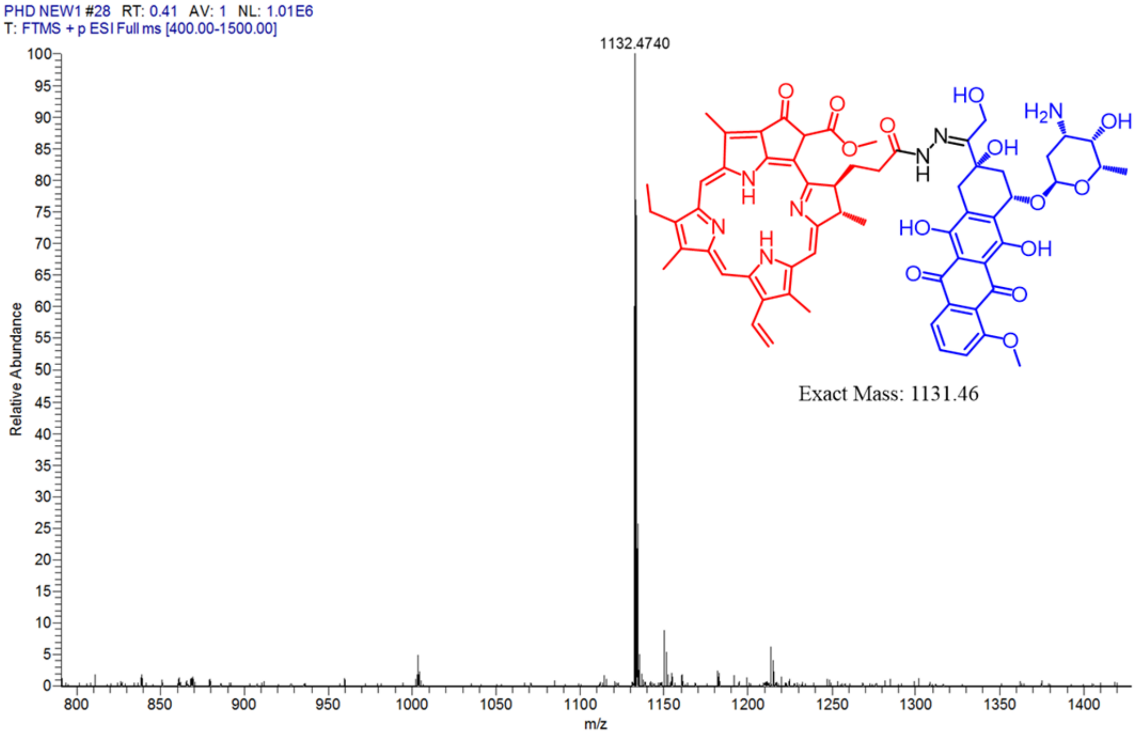

Supplementary Figure 4. HR-MS analysis of molecular weight of PhD. *m/z* 1132.4740 is the M+[H]$^+$ peak.

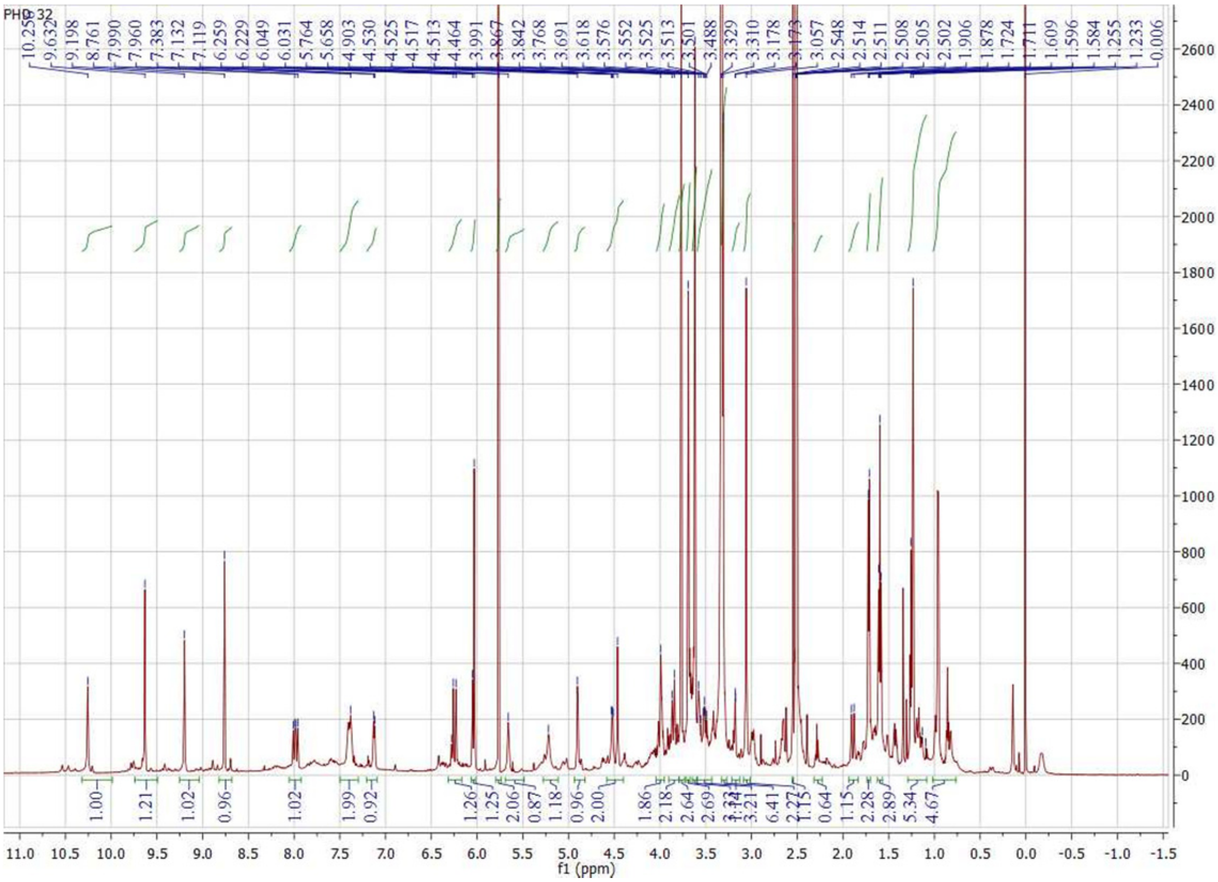

Supplementary Figure 5. The ^{1}H-NMR spectrum of PhD monomer.

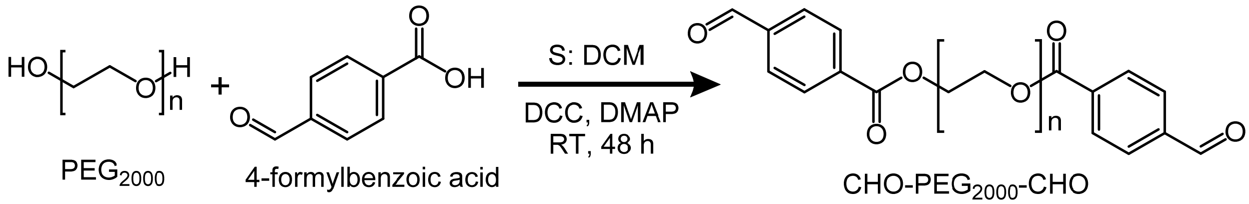

Supplementary Figure 6. Synthetic route of dual-aldehyde terminated PEG_{2000} (PEG-2CHO).

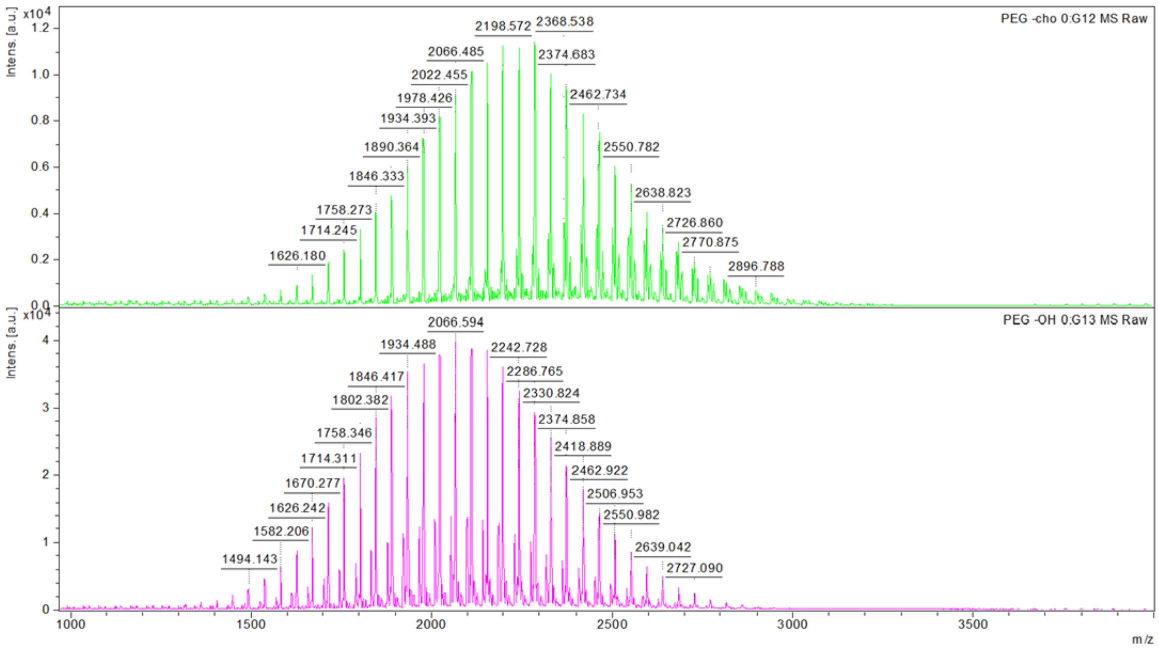

Supplementary Figure 7. Mass spectrometry analysis of PEG-2CHO. The upper panel is PEG-2CHO; the lower panel is the PEG_{2000}. The reaction of aldehyde groups to the PEG caused a molecular weight increase.

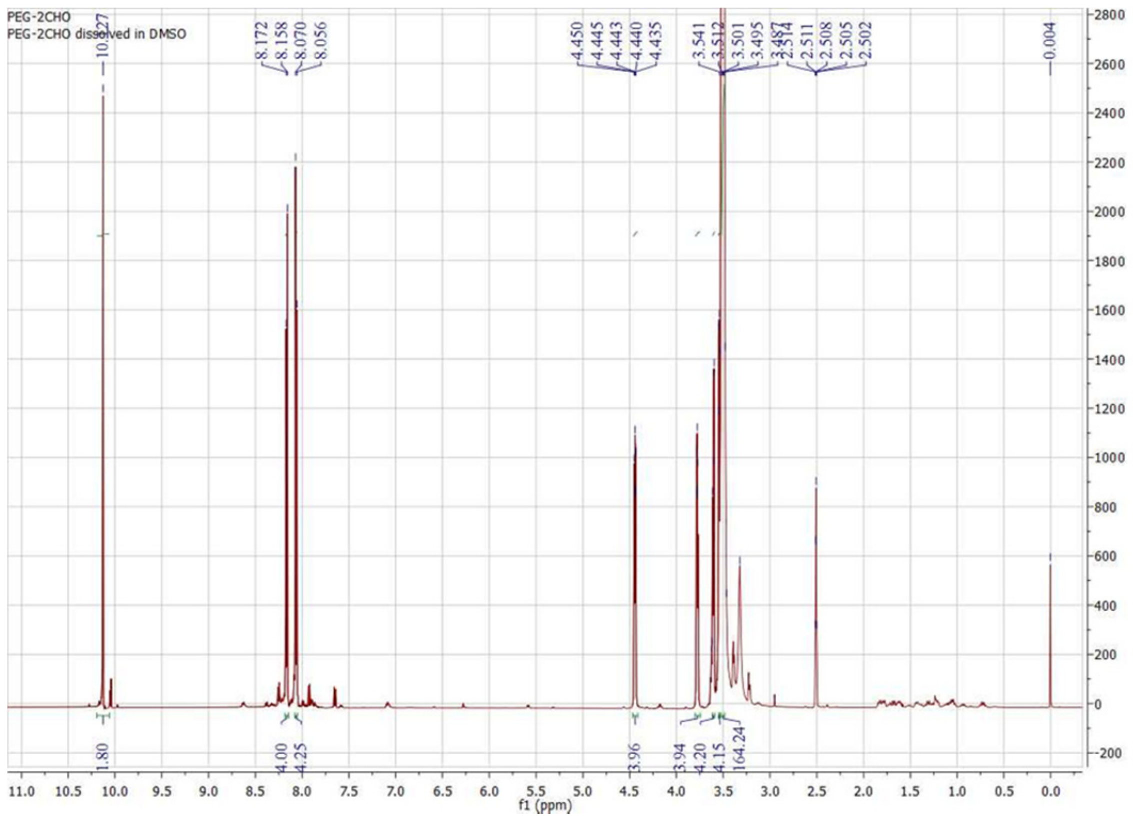

Supplementary Figure 8. The ^{1}H-NMR spectrum of PEG-2CHO.

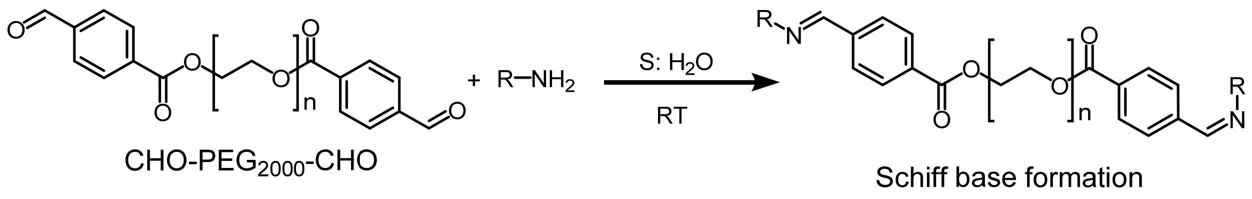

Supplementary Figure 9. The chemical reaction between PEG-2CHO with amine group (Schiff base formation).

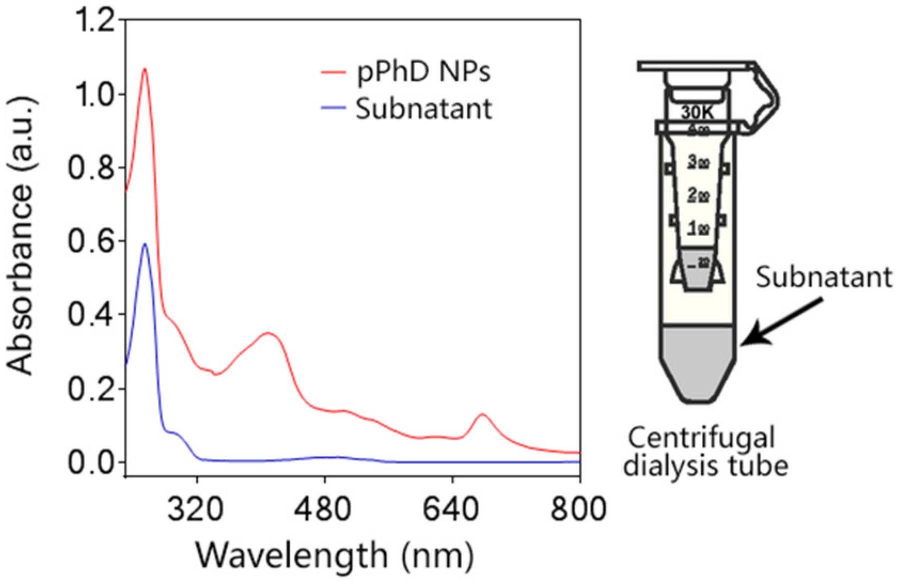

Supplementary Figure 10. The UV-vis absorbance of 100 μM raw pPhD NPs (including 100 μM PhD NPs and 100 μM PEG-2CHO), and the subnatant solution that under the centrifugal dialysis tube after centrifuge. To cut off the nanoparticles only, and remove the free molecules as more as possible, the molecular weight cut-off (MWCO) of the tube was chosen as 30,000 Da. 100 μM PEG was employed to PEGylated 100 μM PhD NPs, and 55 % PEG-2CHO was dialyzed outside of the dialysis tube (free PEG-2CHO that didn't mount to the PhD NPs), which means 45% PEG-2CHO were part of the pPhD NPs. By calculating, the API loading efficiency, including chemotherapeutic drug (DOX) and photosensitizer (Phy), was ~53.3%, in which the drug loading (%DL) was supposed to be ~24.9%, and photosensitizer loading (PL%) is 28.4%.

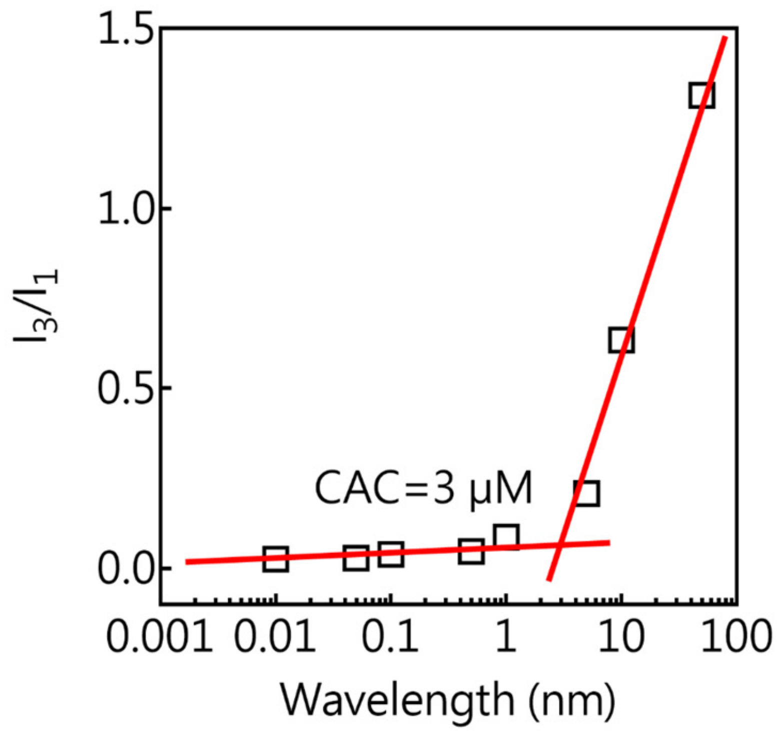

Supplementary Figure 11. The critical aggregation concentrations (CAC) of pPhD NPs measured by using pyrene as a probe.

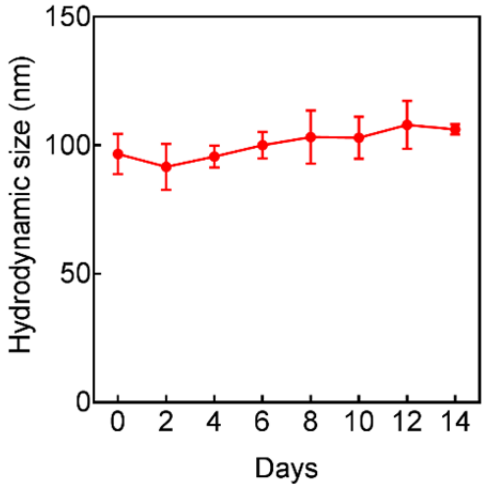

Supplementary Figure 12. Stability of pPhD NPs via measurements of size changes in the presence of serum over time. The pPhD NPs were incubated with 10% fetal bovine serum in 37 ^{o}C. The size distributions were monitored by dynamic light scattering. Error bars=standard deviation (n=3).

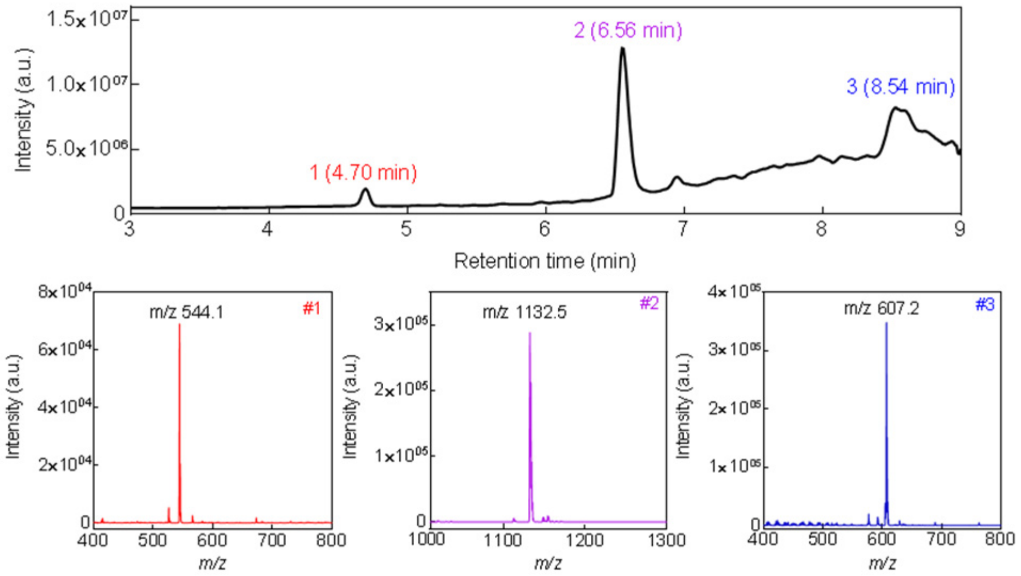

Supplementary Figure 13. The hydrazone bonds cleavage was investigated by liquid chromatography-mass spectrometry (LC-MS). The PhD monomers were incubated in acidic pH (5.0) for overnight and subjected to LC-MS analysis.

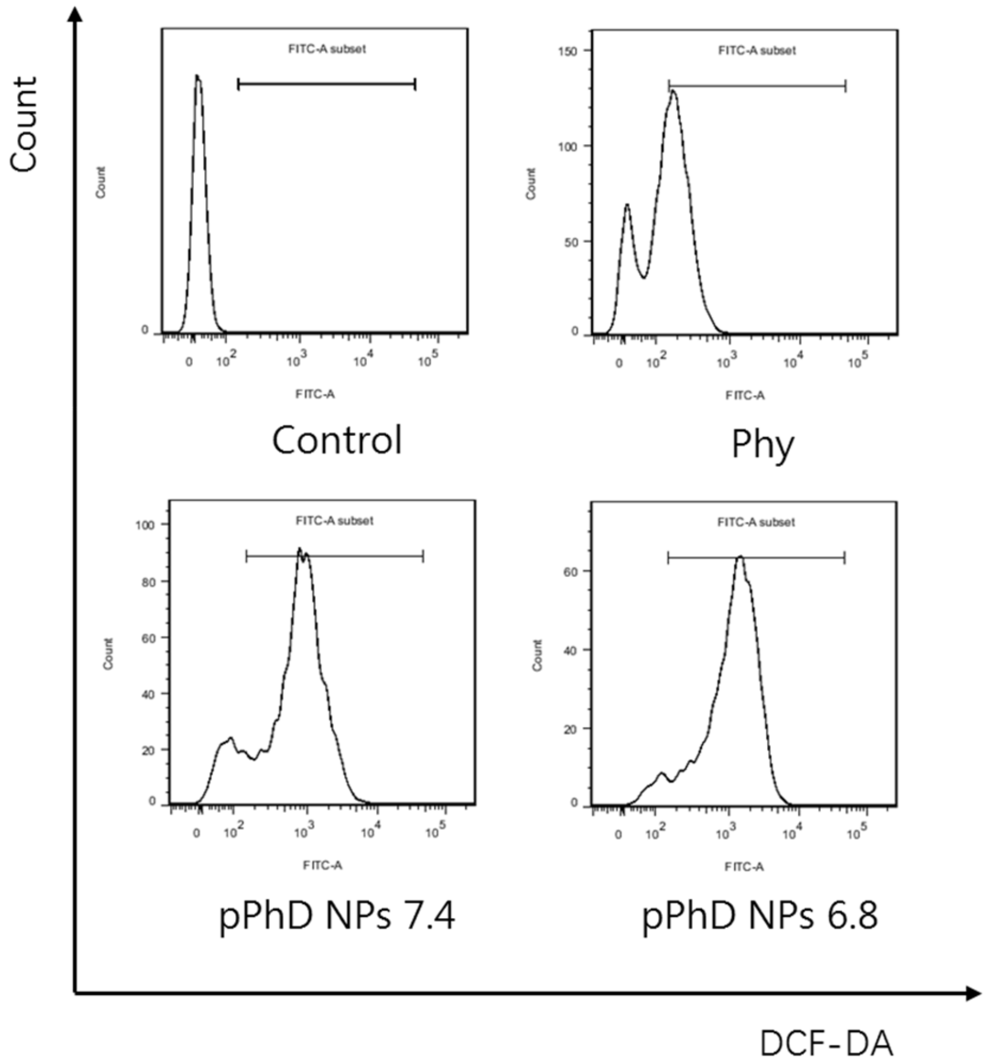

Supplementary Figure 14. Flow cytometry analysis of reactive oxygen species (ROS) production in OSC-3 cells. The intracellular ROS production was indicated by DCF-DA. 5 μM of DOX, Phy, pPhD NPs (pPhD NPs 7.4) and pPhD NPs pre-treated with pH 6.8 (pPhD NPs 6.8) were incubated with OSC-3 cells for 3 h respectively, then applied for light treatment for 1 min. The ROS production was indicated by DCF-DA.

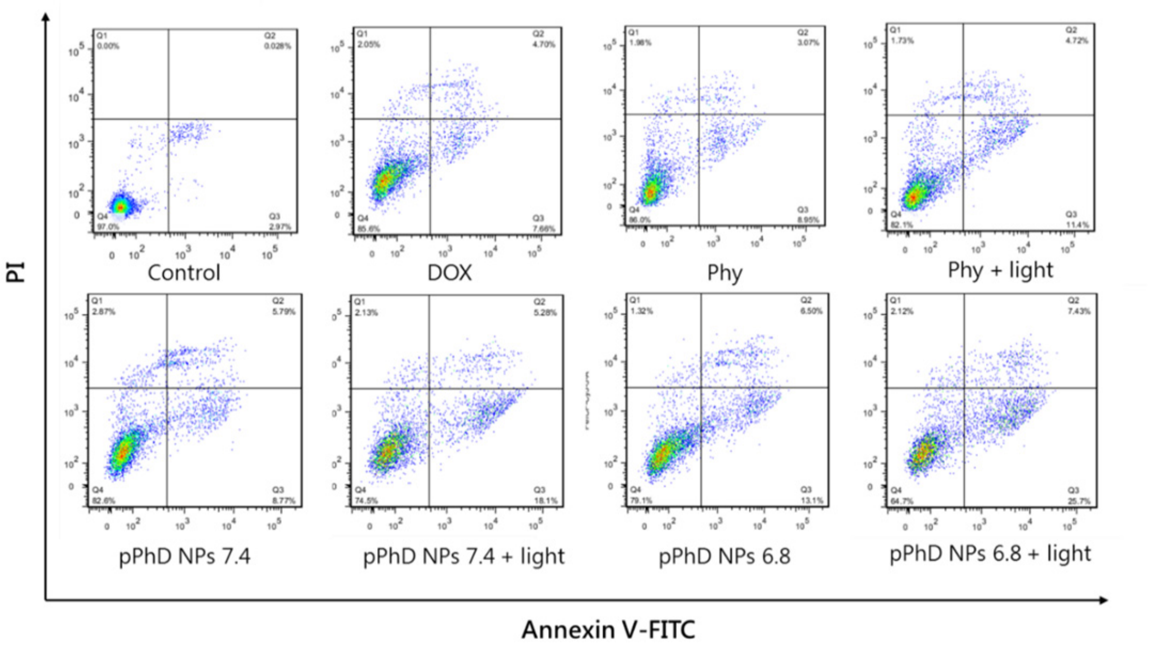

Supplementary Figure 15. Cell apoptosis induced by our materials (all set as 10 μM), including non-treated control, free DOX, free Phy, Phy treated with light, pPhD NPs under neutral pH (pPhD NPs 7.4), pPhD NPs under neutral pH with light (pPhD NPs 7.4 + light), pPhD NPs under neutral acidic pH (pPhD NPs 6.8) and pPhD NPs under neutral acidic pH with light (pPhD NPs 6.8 + light). The apoptosis was indicated by Annexin V-FITC and Propidium iodide (PI) and quantitated by flow cytometry analysis.

Supplementary Table 1. The combination index (CI) between the phototherapy and chemotherapy in pPhD NPs.

**Total Dose**	**Fa**	**CI Value**
0.2	0.99	1.21400
1.0	0.64	0.42287
2.0	0.145	0.17852
10.0	0.009	0.12856
20.0	0.002	0.09464
100.0	0.00875	1.26170

The CI above 1 denotes antagonistic effect, equals to 1 means additive effect and below 1 stands for synergistic effect. The antagonistic effect in the lowest concentration (0.1 μM) and highest concentration (50 μM) were ascribed to the neglectable efficacy and overwhelming efficacy of the phototherapy alone, respectively.

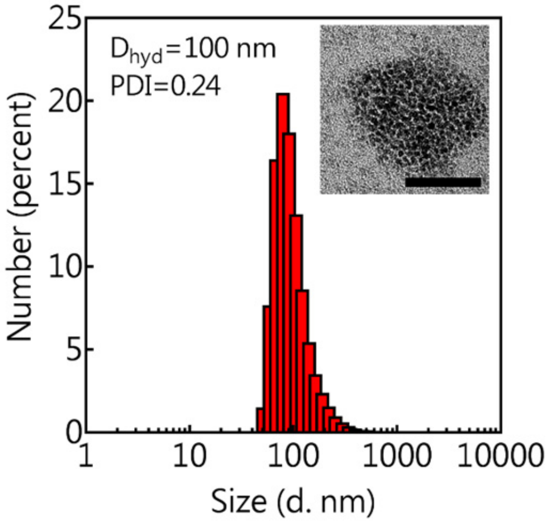

Supplementary Figure 16. The size distribution and morphology of un-PEGylated PhD NPs (upPhD NPs). The scale bar of the inset picture is 50 nm.

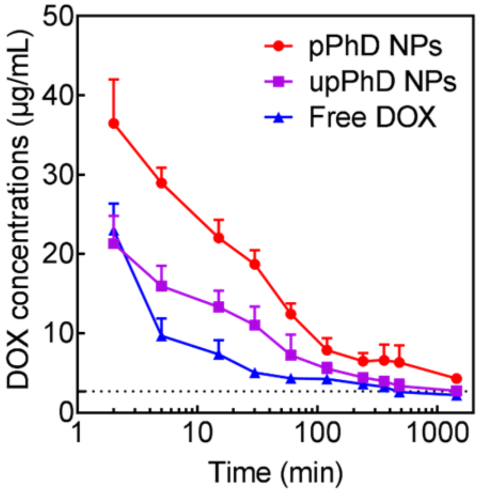

Supplementary Figure 17. Pharmacokinetics of pPhD NPs, upPhD NPs, and free DOX. Error bars=standard deviation (n=3).

Supplementary Table 2. The pharmacokinetic profiles of pPhD NPs, upPhD NPs and DOX.

Group	C-max (μg mL^{-1})	AUC	T-half (α)	T-half (β)
pPhD NPs	36.4744	9258	31.8 min	1440 min
upPhD NPs	21.3182	5505	32.6 min	1095 min
DOX	22.9727	4161	4.4 min	360 min

The C-max, AUC and T-half were calculated by Kinetica 5.0.

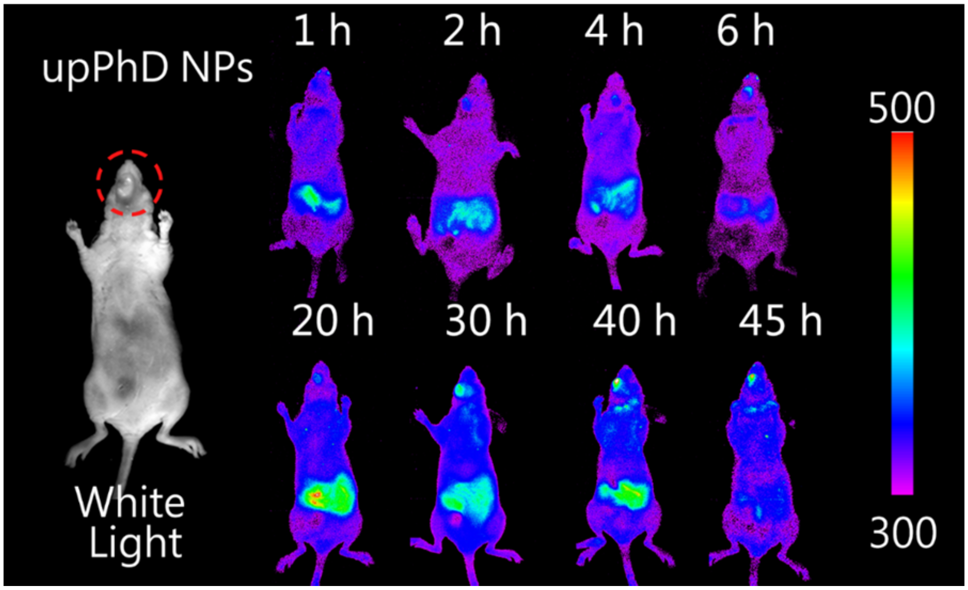

Supplementary Figure 18. Time-dependent, *in vivo* NIRFI of upPhD NPs treated mice. 10 mg kg^{-1} upPhD NPs were i.v. administrated to the mice. The mice were applied for NIRFI at different timepoints. The unit of the gradient bar is given as arbitrary unit (a.u.) to present the fluorescence intensity is relatively higher or lower.

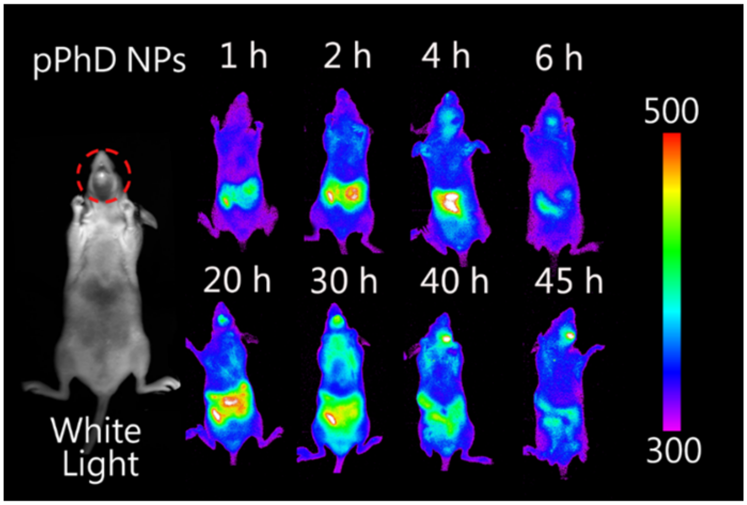

Supplementary Figure 19. Time-dependent, *in vivo* NIRFI of pPhD NPs treated mice. 10 mg kg^{-1} pPhD NPs (calculated based on the concentration of PhD monomer) were i.v. administrated to the mice. The mice were applied for NIRFI at different timepoints. The unit of the gradient bar is given as arbitrary unit (a.u.) to present the fluorescence intensity is relatively higher or lower.

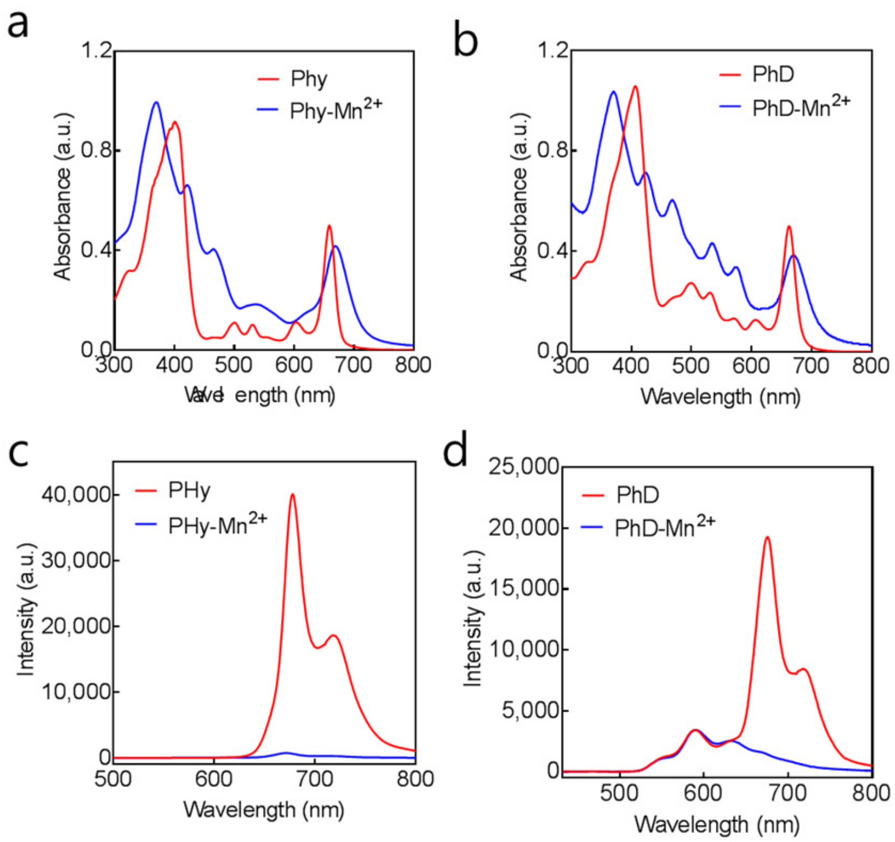

Supplementary Figure 20. Optical evaluation of the Phy and PhD monomer before and after chelated with manganese II (Mn^{2+}). UV-vis absorbance spectra of a) Phy and Mn^{2+} chelated Phy (Phy-Mn^{2+}) and b) PhD monomer and Mn^{2+} chelated PhD monomer (PhD-Mn^{2+}); Fluorescence spectra of c) Phy and Mn^{2+} chelated Phy (Phy-Mn^{2+}) and d) PhD monomer and Mn^{2+} chelated PhD monomer (PhD-Mn^{2+}). The excitation was 412 nm (maximum absorbance of Phy). UV spectra showed that the manganese chelated Phy and PhD exhibited peak shift, the band at 412 nm shift to red, and the one at 680 nm shift to blue, in comparing with their un-chelated counterparts. Fluorescence spectra showed that the fluorescence of Phy were largely quenched, both in manganese chelated Phy and PhD monomers. The optical behaviors supported the successful manganese chelation of our materials.

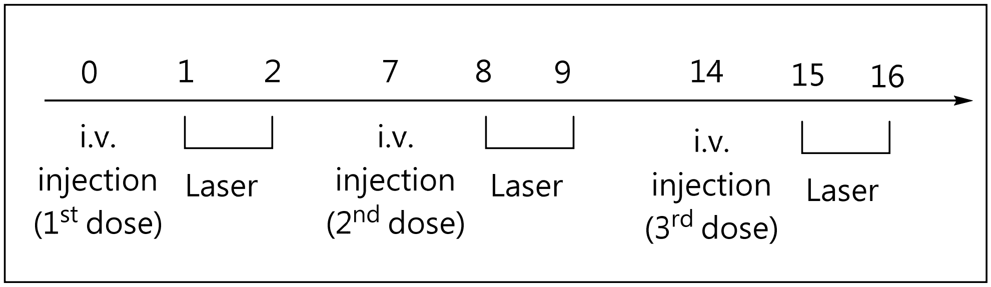

Supplementary Figure 21. Animal workflow. The OSC-3 tumour-bearing mice were randomly assigned to five groups (n=6): control (PBS), free drug (DOX), free photosensitizer (Phy), un-PEGylated PhD NPs (upPhD NPs) and PEGylated PhD NPs (pPhD NPs). The mice received 3 doses of materials via i.v. injection through tail veins. The dose of DOX was 4.7 mg kg^{-1}, Phy was mg kg^{-1}, upPhD NPs were 10 mg kg^{-1} and pPhD NPs were 10 mg kg^{-1} pPhD NPs (calculated based on the concentration of PhD monomer). In subcutaneous models (mice bearing two tumours), the right tumours were subjected to laser exposure (0.4 w cm^{-2}, 3 min), and the left-side tumours were not treated with laser (to evaluate the efficacy of chemotherapy). In the orthotopic models, all the tumours that treated with photosensitizer, including Phy, upPhD NPs and pPhD NPs, were treated with laser (0.4 w cm^{-2}, 3 min). The laser treatments were introduced twice, at 24 h and 48 h after the i.v. injection.

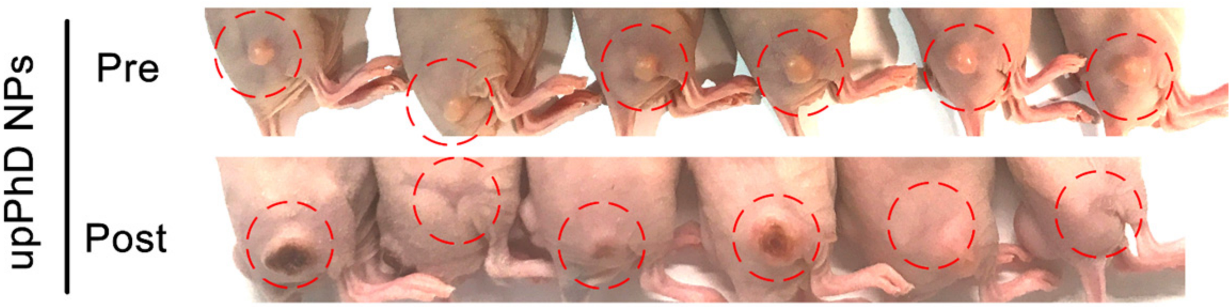

Supplementary Figure 22. The tumour profiles of subcutaneous tumour models (with laser treatment). The red circle pointed out the tumour sites. Pre denotes the mice before the treatment, Post denotes the mice after the 3 doses treatment. The mice were randomly aligned, the upper and lower panel may not correspond to each other.

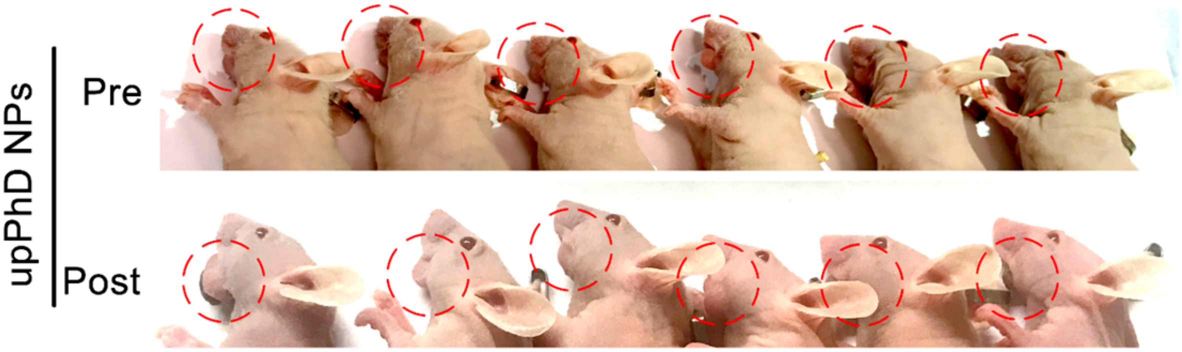

Supplementary Figure 23. The tumour profiles of orthotopic tumour models (with laser treatment). The red circle pointed out the tumour sites. Pre denotes the mice before the treatment, Post denotes the mice after the 3 doses treatment. The mice were randomly aligned, the upper and lower panel may not correspond to each other.

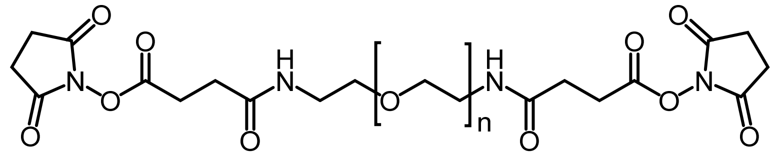

PEG-2COOH NHS ester

Supplementary Figure 24. Chemical structure of PEG-2COOH NHS ester for building the non-transformable pPhD NPs (NT-pPhD NPs).

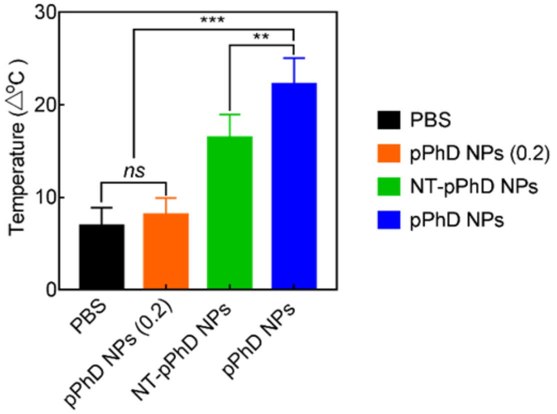

Supplementary Figure 25. Photothermal effect (n=6) of each group on tumour-bearing mice. PBS, NT-pPhD NPs and pPhD NPs were treated with 0.4 w cm^{-2} laser for 3 min; pPhD NPs (0.2) were treated with 0.2 w cm^{-2} laser for 6 min. The PTT experiments were conducted by anesthetizing the mice first, then exposing the tumours under the laser. The body temperature of mice dropped to ~30 ^{o}C due to the anesthesia, and the elevated temperature (lower than 10 ^{o}C) in PBS and pPhD NPs (0.2), therefore, gave fewer efficacies. *ns*, not significant; **, $p<0.01$; ***, $p<0.001$. Error bars=standard deviation (n=3).

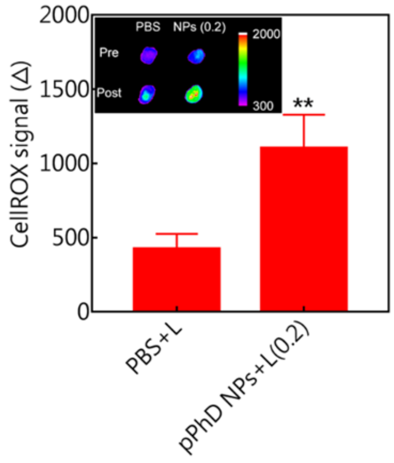

Supplementary Figure 26. Photodynamic effect of pPhD NPs (0.2) in tumours by comparing with PBS (n=3). The mice in PBS and pPhD NPs were both treated with 0.2 w cm^{-2} laser for 6 min. The ROS production was indicated by NIRF ROS probe (CellROX). The inset was the NIRFI of the tumours. **, $p<0.01$. The unit of the gradient bar is given as arbitrary unit (a.u.) to present the fluorescence intensity is relatively higher or lower. Error bars=standard deviation (n=3).

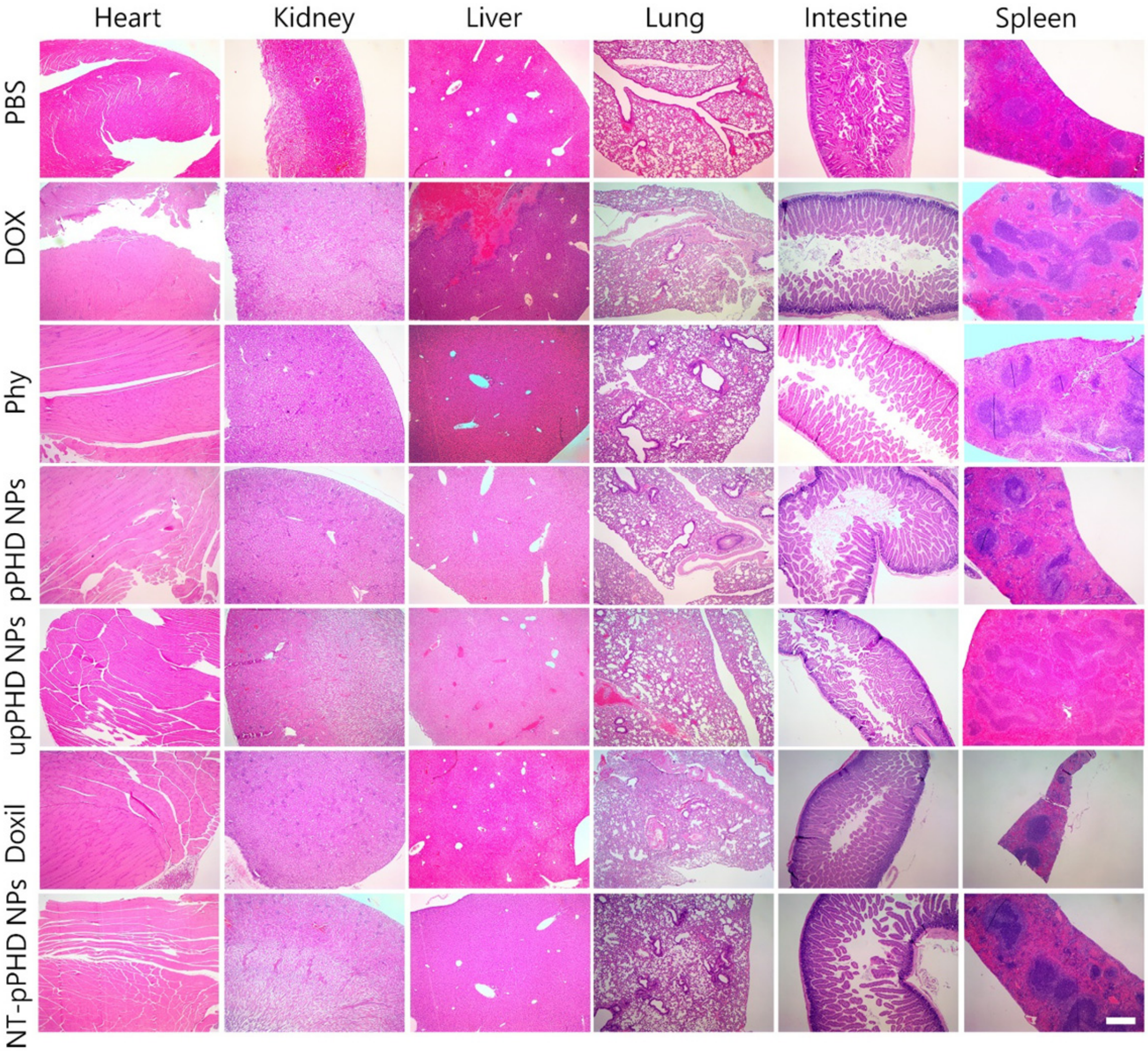

Supplementary Figure 27. H&E stain of main organs illustrated the systemic toxicity of each treatment group in comparison with PBS. The scale bar is 200 μm.
